# Supplementary material for: Are carbon emissions trading and green financial instruments synergistic? -Comprehensive quantitative research based on content analysis
Source: PLoS One. 2024 Mar 7;19(3):e0298601. doi: 10.1371/journal.pone.0298601 (PMC10919601; doi:10.1371/journal.pone.0298601)
Supplement: S1 Data — (ZIP) [file pone.0298601.s001.zip › Data/地级市Z_F工作报告.zip!/┴╔─■/╔≥╤⌠╩╨/└┤╫╘╣1⁄2╓┌║┼í╛┬φ┐╦╩2╛▌═°í┐_2005.docx]

各位代表：

　　现在，我代表市人民政府向大会作工作报告，请予审议，并请各位政协委员提出意见和建议。

　　一、2004年政府工作回顾

　　2004年是沈阳实施中央振兴东北老工业基地战略决策的第一年。在省委、省政府和市委的领导下，在市人大的依法监督和市政协的民主监督下，市政府带领全市人民励精图治，努力拼搏，在振兴沈阳的开局之年旗开得胜，首战告捷。

　　过去的一年，是沈阳经济和社会事业快速发展的一年。全市地区生产总值实现1900.7亿元，同比增长15.5％；固定资产投资完成971.4亿元，增长66.7％；社会消费品零售总额实现808.8亿元，增长12.1％；利用外商直接投资实现24.2亿美元，增长71.7％；财政收入完成138.2亿元（按原口径为181.5亿元），增长33.5％，连续四年保持30％以上的增速，财政支出突破200亿元大关；城市居民人均可支配收入达到8924元，增长12.1％；农民人均纯收入达到4347元，增长13.8％。沈阳各项经济指标的高速增长，引起了国内外的广泛关注，激发了全市人民的万丈豪情。

　　过去的一年，是我市喜事连连、捷报频传的一年。特别是沈阳被评为“2004CCTV全国十大最具经济活力城市”，被国家环保总局命名为“国家环境保护模范城市”，“一宫两陵”被正式列入世界文化遗产名录，“2006沈阳世界园艺博览会”的成功申办，使沈阳在国内外的知名度和影响力有了极大提高，沈阳人终于找回了久违的、属于自己的骄傲和自豪。

　　过去的一年，还是为广大群众办实事最多的一年。全年投入10多亿元，共办了23件实事。对此，广大群众给予了充分肯定，群众的肯定是对我们的最高褒奖。

　　（一）工业经济实现跨越式发展

　　全市规模以上工业总产值实现1493.4亿元，增长36.1％，增幅居15个副省级城市第一位；工业固定资产投资完成278亿元，增长91.8％；工业经济效益综合指数达到127％，上述三项指标都创造了历史新高。第二产业增加值占全市GDP已达49.5％，比上年提高2个百分点，经济结构调整的成效进一步显现。

　　加大了对国有企业改革和改组的力度，出台了包括财政出资三亿元、企业搬迁土地出让金全部用于企业等八项扶持装备制造业发展的政策，重点支持机床等11户优势企业。

　　工业开放度不断提高，去年引进了国外的荷兰东软菲利浦数字医疗、韩国贝尔威夫手机、韩国统一重工汽车车桥和国内的上海通用、三一重工、温州不锈钢等一批大项目。有12亿美元国外资本投向工业。

　　经过近三年的努力，铁西新区成功地闯出了老工业基地改造振兴的新路子。对于铁西新区的巨大变化，国内外朋友给予了充分肯定，全市人民看到了沈阳振兴的希望。

　　经过三年多的建设，浑南高新技术产业开发区已初具规模，浑南新区的迅速崛起，鼓舞了全市人民建设新沈阳的斗志。

　　通过扶持本地民营企业、国有中小企业转制和引进外来民营资本三条途径，促进了民营经济的快速发展。全市民营经济实现增加值1080亿元，占全市GDP的57％，比上年提高4.1个百分点。

　　一年来的实践再次证明，我们促进工业发展的思路是清晰的、正确的；我们迈出的脚步是坚实的、有力的；我们发展工业的条件是充分的、得天独厚的。只要继续努力，沈阳工业就一定能全面振兴。

　　（二）三农工作取得新突破

　　一年来，四县（市）和四个郊区新上规模以上农产品加工企业300家，农产品加工率由29％提高到40％。新建各类富民经济小区697个。粮食总产量达到365万吨，创历史最高水平。累计建设各类种植小区2135个，畜禽、水产养殖小区742个。肉、蛋、奶、鱼产量达到118万吨，蔬菜产量470万吨，水果产量15万吨，花卉种植面积达到三万亩。

　　根据中央一号文件精神，我们制定了包括新增加转移支付2.8亿元等在内的，加速推进四县（市）经济和社会发展的20条措施。四县（市）去年完成生产总值251亿元，同比增长16.6％；完成固定资产投资76.8亿元，增长95.9％；实现财政收入4.6亿元，增长32.3％；农民人均纯收入达到3832元，增长22.7％。法库陶瓷工业园已经签约108条生产线，其中80条已开工建设，14条正式投产；总投资50亿元、装机容量120万千瓦的康平坑口电厂开始“九通一平”，今年将全面开工建设；辽中县的产量100万吨、销售收入60亿元的铸造城已有8户企业入驻，产量50万吨、销售收入100亿元的不锈钢生产基地已经开始建设；新民市丙烯和聚丙烯、20万吨粗铜冶炼和50万吨纸浆、70万吨机制纸三个销售收入过百亿元的项目正在稳步推进。四县（市）经济发展缓慢一直是困扰全市的难题，如今实现了全面突破。

　　四个郊区经济增长创造了历史新高，工业化、城市化进程不断加快。

　　农业高新区主要经济指标成倍增长，一个新经济区的雏型已经出现在沈阳大地上。

　　（三）对内对外开放取得丰硕成果

　　在中央振兴东北老工业基地重大决策鼓舞下，沈阳继上个世纪80年代初的深圳、90年代初的上海之后，成为新世纪国内外投资者聚焦的城市。

　　全市实际调入外资总额居全省第一位，增长71.7％，在全国15个副省级城市中名列前茅。在沈阳投资建厂的世界500强企业已经增加到40家。全市实现进出口总额55亿美元，增长29.1％，其中出口24亿美元，增长19.8％。成功地举办了第三届韩国周、第三届制博会和第二届世界华商企业科技创新合作交流会。

　　东帝汶总统、越南总理、英国副首相等政要来访，进一步提高了沈阳的知名度。市领导会见的外宾达899批10030人次，增长28.6％。桃仙机场旅客吞吐量达到410万人次，增长36.2％。空港出入境人数达到65万人，增长41.3％。全年共接待国内外旅游者2845.1万人次，增长25.2％，其中接待入境旅游者27.1万人次，增长47.3％；旅游外汇收入1.4亿美元，增长27％。全市星级宾馆入住率居高不下。开通了沈阳经北京至法兰克福、洛杉矶和悉尼的三条国际航线。

　　全市先后组织180多个团组、3000多人次，分赴“长三角”、“珠三角”地区开展大规模招商引资活动。全年共引进规模以上项目1707个，总投资额980亿元，已调入资金330亿元。

　　我市申办2006沈阳世界园艺博览会获得成功，全市上下都沉浸在喜庆之中。世园会的举办，将把沈阳的对外开放推上一个新高峰，将大幅度提高沈阳的知名度和影响力，将进一步促进城市整体水平和综合素质的提高，这是世界给予沈阳的一个重大历史机会。

　　（四）环境年活动收到显著成效

　　去年，我们在全市开展了环境年活动，收到了预期的效果。

　　在硬环境建设方面，我们拓展了南北二干线、沈苏快速干道、胜利大街延长路等三条贯通城区南北的大通道，建设了浑河胜利大桥，形成了全市交通的新格局、大格局。完成了覆盖335条二级以下街路、269万平方米的小巷工程；建设了1500公里农村公路，村村通油路已完成96.3％。去年，沈阳荣获了“全国畅通工程模范管理城市”称号。

　　对占道市场和沿街摆卖进行了整治和取缔。非法拼装三轮车营运这一严重影响城市交通及形象的问题，经过大力度整治，取得了阶段性成果。我市进入了全国安静程度最佳城市前10名。

　　全市新增绿地18.7平方公里，建成区绿化覆盖率已达到38.12％，人均公共绿地9.8平方米。我市荣获了“国家园林绿化先进城市”称号。

　　进一步治理了浑河和满堂河10公里的城市段，建设了一批人工湖，全市水面积率由2000年的2.73％提高到去年的4％。由于近几年大规模的绿化和水系建设，使沈阳城区的空气湿度由2002年的51.3％提高到去年的56.9％，正在接近人体最适宜的60％的空气湿度水平。按照我们新制定的水系建设规划，经过几年的努力，沈阳的水面积率将增加到10％左右。沈阳人也要尽情享受水带给我们的舒适、美感和创造力。

　　去年又拆除锅炉房400座、烟囱1003根，使集中供热率达到80％。空气质量优良天数达到301天，再创历史最高水平。经对全市九个水厂实行月度监测，我市成为全国集中式饮用水源水质100％达标城市。

　　三年创模，终获佳绩。沈阳被国家环保总局命名为“国家环境保护模范城市”。一个老工业基地，一个原来严重污染的典型的脏、乱、差城市，能获此殊荣，实属来之不易。这是全市人民辛勤汗水的结晶，也是沈阳城市建设一个新的里程碑，沈阳人民为拥有这样一个日益温馨美好、蒸蒸日上的城市而感到无比自豪。

　　在软环境建设方面，行政审批事项在前年削减至239项的基础上，去年再次削减至165项，成为全国大城市中行政审批事项最少的城市。完成了市政府机构改革任务，调整了58项政府职能。全年坚持开展了每两周一次的市长接待日活动，共举办22次，已解决201件企事业单位发展过程中遇到的难点问题。全市132项年检项目已减少28项，延长时限40项，占原有项目的51.5％。

　　向广大市民征集有关环境建设的意见建议1909条，并有选择地进行了吸纳。文明出行、文明祭祀等活动得到热烈响应，清明节等传统节日烧纸祭祀的现象明显减少。

　　（五）第三产业日益繁荣

　　南北金廊建设取得可喜进展。规划区内的在建项目已达32个，建筑面积465万平方米，总投资额245亿元。一个中央都市走廊的雏型已经形成，一个城市的脊梁将在这里挺起。

　　去年5月，正式启动了城市段长42公里、面积171平方公里的浑河两岸开发。目前已有26个项目落地，实际投资已达158亿元。浑河两岸基础设施建设全面启动，已建成8.2公里的大堤路和32公里的滨水路，实施了32公里城市段滩地综合整治。浑河东西银带的开发建设，不仅与南北金廊共同构成了未来沈阳城市发展的大十字新格局，同时还将把沈阳建设成一个拥有美丽内河的大都市。

　　棋盘山旅游开发区是继浑南新区、铁西新区、农业高新区、金廊和银带之后，沈阳规划开发的第六大发展空间，其面积已由73.5平方公里调整为190平方公里，并将以世园会为契机，拉开沈阳东部大开发的序幕。

　　城乡商业购销两旺，全市5000平方米以上的大型综合超市和专业超市达到42家，其中10000平方米以上超市31家；连锁店铺总数达4000余个。

　　全年商品房销售面积达到770万平方米，交易金额245.2亿元，同比分别增长48.6％和48.5％。沈阳已经成为国内房地产市场最活跃、最规范的城市。地产、房产的大幅度升值，带动了市民财富的增加和城市价值的提升。

　　金融机构存贷款余额分别达到3206亿元和2371亿元，同比分别增长7.5％和7.8％。沈阳设立了第一家外资银行———韩国第三大银行韩亚银行分行。万顺达集团公司成功地在新加坡联交所挂牌上市。

　　（六）社会保障体系进一步完善

　　全市参加养老保险的从业人员增加到131万人；参加医疗保险的人员增加到175.4万人；参加失业保险的人员达到118.5万人；全市有16.3万人享受低保；最低工资标准由原来的320元上调到400元；城市低保标准由205元调整到220元。

　　去年，将企业养老保险费征缴比例由23.5％下调到20％，每年可为企业减轻负担3.5亿元；将企业退休职工统筹项目外的49元，全部纳入统筹项目内发放，每年又可为企业减轻负担近四亿元。这是我们过去多年想办而难以办到的两件事，它不仅减轻了企业负担，改善了投资环境，同时也将有力地促进养老保险面的扩大。

　　全年开发就业岗位15.2万个，实现就业和再就业22.4万人次，培训下岗职工8.6万人次。对“4050”大龄下岗人员，我们采取积极措施，只要本人有就业要求，就保证安排公益性岗位。帮助这部分人员就业，是政府的重要责任。全年共安排2.1万人走上了公益性岗位。城镇登记失业率为5.7％。APEC劳动保障高层研讨会的各成员代表，在对我市劳动力市场和社保经办机构考察后，给予了高度评价。我市被国务院授予“全国再就业先进工作单位”荣誉称号。

　　（七）社会事业全面发展

　　制定并实施了《振兴沈阳老工业基地十大科技行动纲要》。全市有87个重点科研项目进入国家863、科技攻关等计划，举办重大科技活动32项。创建农民科技培训基地12个。全年实现高新技术产品产值900亿元，同比增长18.9％。芯片、网络可视电话等一批处于世界领先水平的新项目落户沈阳，推动了产业升级和结构调整。

　　全市农村合并了158所小学，建设了11所九年一贯制学校，改造116所中小学危房，培训了1000名教师。基础教育改革试验区工作取得阶段性成果，22所改革试点学校在校舍建设和师资水平上有较大提高；教师人事制度改革进一步深化。全市有16所学校被确定为省级示范高中。职业教育得到了进一步加强。全年从国内引进和培养各类人才4.2万人，引进国外专家2200多人次。

　　成功举办了“中国·沈阳香格蔚蓝新春灯会”、“清文化节”、“纪念锡伯族西迁240周年”、首届龙舟大赛、户外体育嘉年华和万人健康跑等系列活动。舞剧《天祭》荣获国家文化部第11届“文华新剧目奖”。文化体制改革试点工作稳步推进，持续开展“网吧”专项整治行动，加强了文化市场的建设与管理，“扫黄”、“打非”取得了新成果。

　　投资五亿元建设的四大文化设施中，残疾人康复教育培训中心已经投入使用，少年儿童活动中心、市图书馆和市档案馆将于今年相继建成。特别是去年7月1日，“一宫两陵”正式列入世界文化遗产名录，为此全市一片欢腾，沈阳终于有了享誉中外的靓丽名片。

　　在法库县开展了以大病统筹为主的新型农村合作医疗制度的试点。实施了《沈阳市公共卫生体系建设规划》，完善了公共卫生体系。加强对重大传染病的预防与控制，有效遏制了各类急慢性传染病的发生和蔓延。加强了农村医疗服务体系的建设。

　　沈阳籍运动员在第28届奥运会上取得历史最好成绩。市35中学荣获亚洲中学生排球赛亚军，市31中学荣获全国高中生篮球联赛冠军。在350个社区配置了健身器材，社区健身器材普及率达到70％。

　　投资1.2亿元，解决了县区40万群众饮用高氟水问题；投资5000万元，改造24条街路、102处积水点和12座泵站；筹资2400万元，资助4.4万名贫困家庭学生；新年春节期间向2.2万户贫困农民捐赠了彩电；为低保户和特困群体支付1.2亿元采暖补贴和2600万元医疗费；为市民补贴取暖费1.2亿元；为8000户困难户提供政府补贴房40万平方米；安装315条无灯街的路灯，并对130条照明度不够的街路更换灯具。市委、市政府一直把群众的困难挂在心上，解决群众的困难和问题，永远是我们的工作重点。

　　（八）依法治市不断深化

　　去年，向市人大提报了《沈阳市社会保险费征缴条例》等三件地方性法规草案、《沈阳市体育设施管理条例》等五件地方性法规修改草案，以及《沈阳市乡镇企业条例》等五件地方性法规废止案。市政府制定了《沈阳市城市房屋拆迁管理办法》等九件政府规章。全市认真贯彻实施《行政许可法》，深入开展行政执法监督检查工作，着力解决乱检查、乱收费、乱罚款问题。

　　市政府自觉接受人大依法监督和政协民主监督，坚持定期向市人大常委会主任会议和市政协主席会议通报工作情况。全年办理人大代表建议和政协提案1733件，办复率达100％。政府自身建设进一步加强，抓细节、求实效的风气正在形成。

　　严厉打击了境内外敌对势力和“法轮功”等邪教组织的破坏活动，深入开展了校园及周边治安秩序整治等专项行动。市政府投资4600余万元，公安机关组织3300余名警力，在市区实行了全时空覆盖的“网格化”巡逻，使刑事案件发案数比去年下降14.7％，在全国大城市中是下降幅度最大的。

　　依法加强信访工作，妥善处理和化解大量热点、难点问题。积极推进政府机关的勤政廉政建设，重点开展了教育乱收费、减轻农民负担等专项清理工作，纠风治乱工作取得了新进展。

　　社会公共安全形势平稳，近年来市财政投入2.56亿元加强消防工作，使沈阳消防设施水平跃居国内副省级城市前列。各级党政组织多次组织拉网式安全生产大检查，对各类安全隐患进行严格整改。去年全市共发生各类事故8706起，同比下降0.07％；死亡812人，同比下降3.33％；直接经济损失3580.1万元，下降11.5％。

　　人口与计划生育、统计、审计、物价、参事、民族、宗教、广播、电视、新闻、出版、侨务、质量技术监督、药品监督、民兵、预备役、人防、档案、修志、文史、残联、红十字会和老龄工作也都取得了新成果。

　　各位代表，在回顾和总结去年工作的时候，我们深知，一年所取得的各项成绩，无不凝聚着全市人民的心血和汗水，无不体现着各方面的帮助和支持。在这里，我代表市政府，向为沈阳发展做出无私奉献、付出辛勤劳动的全市各族人民致以亲切的问候和崇高的敬意！向给予我们支持与监督的人大代表、政协委员，各民主党派、工商联和无党派人士、人民团体，向参与沈阳发展建设的中央和省在沈单位、驻沈部队指战员、武警官兵和在沈工作的外籍人士、港澳台同胞、海外侨胞及国际友人表示衷心的感谢！

　　在充分肯定成绩的同时，我们应当看到，当前还存在一些亟待解决的矛盾和问题，主要表现为：一是工业经济总量偏小，缺少产值在百亿元以上的大型企业；二是经济外向度低，出口总额少；三是经济发展环境尚存在不少问题，政府机关的工作作风、业务水平有待进一步改进和提高；四是目前社会还存在一些不稳定因素，一些群众的生活还存在不少困难。我们要高度重视这些问题，在今后的工作中，采取有力措施，切实加以解决。二、2005年政府工作安排

　　今年政府工作的指导思想是：坚持以邓小平理论和“三个代表”重要思想为指导，认真贯彻党的十六届三中、四中全会和中央经济工作会议、省委九届八次全会和市委十届十次全会精神，以科学发展观为统领，以改革开放为动力，以“提高振兴能力，加快先行步伐”为主题，以“工业年”活动和世园会筹备为载体，加速优化经济结构，坚持“工业立市”，切实转变经济增长方式，全面提升城市品位，不断完善城市功能，积极推进“大沈阳”建设，带动沈阳经济区的发展。大力加强精神文明、民主法制建设，努力构建“和谐沈阳”，不断开创振兴老工业基地的新局面。

　　今年全市经济和社会发展的主要预期目标是：地区生产总值增长12.5％，财政收入增长15％，固定资产投资增长15％，利用外商直接投资增长20％，社会消费品零售总额增长11％，城市居民人均可支配收入和农民人均纯收入分别增长11％和9％。

　　（一）紧紧围绕工业年活动，加速老工业基地调整改革步伐

　　为了加速振兴沈阳老工业基地，为了使全市上下聚焦工业，为了充分利用难得的发展工业的各种有利条件，市委、市政府决定今年在全市开展“工业年”活动，并制定了《沈阳市开展“工业年”活动实施方案》。“工业年”的总体目标是：2005年全市规模以上工业总产值要过2000亿元大关。2007年和2010年全市工业要再迈上两个新台阶。

　　坚持走新型工业化道路。通过对现有企业的整合，培育20个销售收入过百亿元的大企业；通过对国内经济发达地区招商，引进20个左右销售收入过百亿元的企业集群；通过对国外的开放，引进20个销售收入过百亿元的大企业。

　　财政要筹集20亿元，用于支持工业的发展。办好一批装备制造业类职业技术学校，为全市工业发展提供人才支持。做好资金、运输和原材料等多方面的协调服务，为工业企业生产经营创造良好条件。全年要争取有五家企业在境外上市，加速筹集发展资金。

　　继续推进国有工业企业的战略性重组。全面推进国有大型企业的股份制改造；完成国有中小企业的转制收尾工作；对“问题企业”和“壳企业”分期妥善处理。进一步推进国有资产管理体制改革，加强监管，确保国有资产保值增值。继续大力发展民营经济，发展各类社会中介组织和机构。

　　我们要下定决心，集中力量，努力拼搏，务求实现“工业年”的各项目标，搭建起沈阳工业的新框架。

　　（二）筹备好世园会，全面提升城市水平

　　广泛借鉴世界各国的经验，细致扎实地做好世园会的各项筹备工作。世博园的90余个展园、三个展馆以及一门、一广场和一标志性建筑的建设，必须在国庆节前全部完工。对世园会景区内的九条公路进行全面改造，对东陵路、沈吉铁路沿线进行全面整治和绿化。按照体现自然生态的要求，对浑河滩地、岛屿等五平方公里进行整治和建设，构筑世园会的外景平台。

　　以世园会为契机，今年要全面启动190平方公里的棋盘山旅游开发区规划和建设，引进和建设一大批旅游度假项目。

　　全市人民要万众一心，克服一切困难，用最新、最美的创意，争取把2006沈阳世界园艺博览会办成国际一流的园艺博览盛会，在世界园艺史上留下浓墨重彩的一笔。世界给沈阳一个机会，沈阳一定要还世界一个奇迹！

　　要以筹备世园会为契机，全面提升城市建设和管理水平。重点做好北陵大街等16条主要街路两侧的绿化，小区内的拆违建绿和庭院绿化；在中心城区新建100块街头公共绿地，新增绿地八平方公里，建成区绿化覆盖率要达到40％。

　　改造二环路和青年大街等八条干线道路，整修道路总面积140万平方米；进一步完善道路标识、地下通道、安全岛等功能设施；继续实施小巷工程，今年要整修420条小巷街路，总面积230万平方米；开工建设沈阳至康平的高速公路；完成1000公里农村公路建设，全面实现村村通油路；新增公交车500台。要加强对城市地下空间的规划利用，继续积极争取国家批准地铁项目。

　　启动水系建设工程，对辉山明渠、724明渠、满堂河进行全面整治，在城市东部形成三条水生态景观带；扩建丁香湖，建设东湖，使全市水面积率提高到5％左右。

　　今年要再拆除烟囱1000根，拆除10吨以下锅炉房600座。

　　全面落实沈阳市城市管理工作会议精神，加大综合整治力度，继续拆除市区内的违章建筑，推进户外广告设置的市场化进程。深入开展爱国卫生运动，争创“国家卫生城市”。同时，启动我市生态城市创建工作。

　　（三）大力发展县和郊区经济，加快推动城市化进程

　　继续落实好关于加快县域经济发展的20项政策措施，加快农业产业结构调整的步伐。今年要新建畜牧小区150个，畜牧业占农业产值的比重达到50％。新增花卉种植面积二万亩、苗木培育面积一万亩。全市粮食播种面积稳定在550万亩，粮食产量稳定在280万吨。

　　要加大对农业的投入力度，大力发展设施农业、订单农业、加工农业、观光农业等现代农业，积极推行农产品标准化，在提高农产品质量上狠下功夫。

　　今年继续给县城和苏家屯区、新城子区各1000万元建设资金，继续给各县（市）工业小区基础设施建设资金1000万元，支持县区发展。要进一步开展村屯环境整治。

　　今年全部取消农业税。确保全年化解村级债务的10％。新型合作医疗要在全市农村全面铺开。全年争取实现农村劳务输出20万人。

　　农业高新区要继续保持快速增长势头，主要指标要努力做到成倍增长。

　　要把郊区工作进一步提上重要议事日程，年内要召开郊区工作会议，采取更有力的措施，加快郊区城市化进程。今年将在适当时机启动沈北大开发。

　　（四）加大对外开放力度，进一步提高国际化程度

　　要多渠道、宽领域、大力度地推进对外开放。继续办好制博会、韩国周等大型活动，办好第三届中日经贸合作会议、日本周等国际活动。力争每月举办一次大型活动，使各种展会和经贸活动接连不断，搭建起与国际全面对接的各种平台。

　　利用外商直接投资要力争达到32亿美元，外贸出口力争达到30亿美元。积极争取更多的外国元首和政要来沈阳访问。新开通国际航线三条，空港出入境人数要增长30％以上。

　　要抓住国内南资北上的机遇，以温州和闽南为重点，继续吸引更多的资金和人才进入我市。

　　（五）加速发展现代服务业，进一步拓展中心城市功能

　　加快南北金廊的开发建设步伐，争取有十个新项目落地，努力建设好这条中央都市走廊。

　　加大浑河两岸开发建设的力度。在继续做好基础设施和绿化美化的同时，今年要力争新开工20个项目。

　　推进太原街、中街、长江街、东城商业区、北市场和西塔街等繁华商业街区的建设和改造。统一规划、合理布局，积极发展连锁经营、专卖店、超市和现代物流配送业。

　　要积极开展区域联合，整合旅游资源，今年外地来沈旅游人数要比去年增加500万人次；全市旅游业收入要增长25％。

　　努力吸引国内外银行、保险、证券和风险投资等机构在我市设立分支机构，加速沈阳区域市场金融保险业的发展。住房消费贷款余额要突破350亿元，比上年增长30％以上。继续办好春秋两季的房产交易会，进一步活跃房地产市场。

　　（六）提高城市文化品位，推动社会各项事业协调发展

　　进一步推进文化体制改革，通过转换机制，面向市场，拓展服务功能，促进文化事业和文化产业的健康发展。继续举办新春灯会，积极筹办好世界杯自由式滑雪沈阳站比赛、国际龙舟大赛和杂技大赛等系列活动。

　　加强对全市历史文化遗产和文物的保护。对占地1.7平方公里的皇城要做出整治规划，逐步实施，使之成为更有名气的旅游胜地。对北市地区进行全面规划，建设大剧院、杂技厅、露天剧场和商业街等设施，使之成为更具娱乐、商业特色的城市空间。

　　加速科技事业的发展，全市高新技术产品产值要争取实现1100亿元，同比增长20％以上；高新技术产品出口要达到15亿美元，增长30％以上；建设农民科技研修基地100个；大力扶持和发展民营高科技企业。

　　努力提高教育质量，改善薄弱学校的办学条件，推进教育均衡发展。全市农村今年将建设15－20所九年一贯制学校，改造校舍危房2.3万平方米，为农村培养骨干教师1000名。农村初中学生辍学率要控制在3％以内。大力发展职业教育，争取省级示范中等职业学校达到20所，其中15所达到国家级重点职业学校标准。

　　全面完成《沈阳市公共卫生体系建设规划》，建立起运转协调的公共卫生体系。加强农村卫生医疗服务体系的建设，加强对乡镇卫生院的建设，改善农村基础医疗条件。

　　充分发挥我市科研院所和高等院校的作用，积极引进

　　国内外科技及各类人才，为老工业基地振兴提供人才智力保障。

　　搞好特色体育活动，要在300个社区安装健身器材。办好沈阳市第11届体育运动大会。继续做好2008年北京奥运会足球分赛场的宣传和场馆建设工作。

　　（七）扎实推进社保扩面和就业再就业工作，不断完善社保体系

　　今年社保工作的重点是做好养老保险的扩面工作。劳动者参加养老保险，不仅关系到社保资金的筹集，更关系到老有所养的大问题。因此，我们必须对所有参加工作的人员都做到应保尽保。医疗保险要达到180万人，同时确保退休人员社会化管理、档案移交社区和个人帐户做实“三个100％”。

　　稳步推进就业和再就业工作。“4050”人员是今年再就业工作的重点，不仅要保证为这些人提供公益性岗位，同时，还要落实养老保险政策。全年要力争实现社区就业9万人，城镇登记失业率控制在6％以内。要争取为再就业人员发放一亿元的小额贷款。

　　市内要新建两处大型劳动力市场，推动就业和再就业工作。重点做好就业前的培训工作，今年要培训八万人次。

　　建立农村低保制度，将现有农村8.6万贫困人口和其他应纳入低保线的全部纳入低保范围。

　　继续做好扶贫帮困工作，尤其要对贫困残疾人提供重点帮助。全年已确定的17项扶贫工作，每项都必须落到实处。我们要采取各种举措，使财政支出进一步向低收入群体倾斜。

　　（八）加强依法行政，建设廉洁、勤政、高效政府

　　全市各级政府要自觉接受人大的依法监督和政协的民主监督，认真办理人民代表的议案、建议、批评和意见，认真办理政协提案，充分听取各民主党派、工商联、无党派人士和人民团体的意见建议。要继续完善职工代表大会和村民自治等制度。要认真制定好全市“十一五”国民经济和社会发展规划。

　　深入贯彻实施国务院《全面推进依法行政实施纲要》，建设法治政府。不断完善对政府公务人员的监督制度，加强干部队伍的作风建设，进一步牢固树立干实事、求实效，做深入细致工作的良好风气。要继续深化行政审批制度改革，坚持举办好“市长接待日”，使之真正成为政府与企业、市民密切联系的“绿色通道”。

　　要加强社会公德教育，努力建设诚信社会。我们的政府，要建成诚信的政府；我们的企业，要建成诚信的企业；我们的市民，要成为诚信的市民。要开展国防教育，增强全民国防意识，加强预备役部队建设。

　　要按照市委十届十次全会精神，在各级政府认真开展“保持共产党员先进性教育”活动，通过加强党的自身建设，不断开展反腐败斗争。充分发挥党员的先锋模范作用，推动政府的各项工作，提高执政能力。

　　（九）坚持执政为民，为群众多办好事实事

　　要坚持执政为民的根本宗旨，关注困难群体，积极做好扶贫帮困，推进城乡新型社会救助体系建设，发展社会福利和慈善事业。要通过发展生产等切实措施，不断提高人民群众的生活水平。

　　要继续大力开展为群众办实事活动，要围绕就业、医疗、住房和文体活动等方面，努力办好22件实事。

　　要从代表人民群众根本利益的高度，千方百计抓好社会公共安全。各级政府要把安全生产、消防安全和应对突发灾害纳入重要日程，确保广大市民的生命财产安全。

　　要继续贯彻打防结合、预防为主的方针，进一步完善社会治安防控体系，坚持“网格化”巡逻等行之有效的措施，严厉打击各种刑事犯罪活动，确保社会治安稳定。进一步做好各级信访、市民投诉和社区人民调解工作，妥善处理各种突发性的群体事件，及时有效地化解各类人民内部矛盾，加快构建和谐沈阳。

　　各位代表，沈阳所面临的重大历史发展机遇千载难逢；全市上下政通人和、士气高涨的局面前所少见。让我们在市委的领导下，万众一心，乘势而上，努力拼搏，去创造更加灿烂的明天！
